# Increased ABCC2 expression predicts cisplatin resistance in non‐small cell lung cancer

**DOI:** 10.1002/cbf.3577

**Published:** 2020-08-20

**Authors:** Yun Chen, Hongying Zhou, Sifu Yang, Dan Su

**Affiliations:** ^1^ Department of Oncology Zhejiang Provincial People's Hospital, People's Hospital of Hangzhou Medical College Hangzhou China

**Keywords:** ABCC2, apoptosis, cell cycle, cisplatin‐resistance, NSCLC

## Abstract

Long‐term use of platinum‐based drugs can cause non‐small cell lung cancer (NSCLC) to develop extremely strong drug resistance. Increasing the drug dosage does not have better treatment effects and could lead to serious complications. High levels of drug resistance are considered to be characteristic of human tumours and are usually mediated by genes related to multidrug resistance. Multidrug resistance‐associated protein 2 (ABCC2), an ATP‐binding cassette multidrug resistance transporter, was found to be overexpressed in various human cancers. In this study, we found that ABCC2 was also upregulated in cisplatin (DDP)‐resistant A549 cells (A549/DDP). Functional studies demonstrated that ABCC2 knockdown reversed DDP resistance and promoted G1 phase arrest in A549/DDP cells, and PARP and caspase‐3 were activated in A549/DDP cells following ABCC2 knockdown. In vivo, ABCC2 knockdown enhanced the cytotoxicity of DDP to subcutaneous A549 tumours. Together, these results suggest that ABCC2 may be a potential therapeutic strategy for overcoming DDP resistance in NSCLC patients.

**Significance of the study:**

In this study, we investigated the role of ABCC2 in cisplatin resistance of NSCLC cells. Our data show that ABCC2 expression was associated with resistance to cisplatin and that knockdown ABCC2 could reverse cisplatin resistance in NSCLC cells. Taken together, our study suggests that reducing the expression of ABCC2 could become an important strategy for enhancing the sensitivity of NSCLC cells to cisplatin.

## 1 INTRODUCTION

Lung cancer has a high incidence and strong metastatic ability. It includes small cell lung cancer and non‐small cell lung cancer (NSCLC). Although NSCLC is not as malignant as small cell lung cancer, nearly 90% of lung cancers are NSCLC.^1^ Cisplatin (DDP) was first synthesized by M. Peyrone in 1845, and subsequently proved effective at killing NSCLC cell, and was approved for use by the FDA in 1978. For NSCLC patients, adjuvant chemotherapy based on DDP is usually recommended after surgical resection of the tumour. However, when DDP is used for a long time, most patients will become resistant to DDP and the NSCLC can no longer be controlled. Previous studies have demonstrated drug resistance mechanisms in cells, including enhanced DDP cell detoxification, inhibition of apoptosis, and enhanced DNA repair capabilities.^2^, ^3^ These drug resistance factors significantly reduce the efficacy of lung cancer treatment in patients. Therefore, it is necessary to study platinum drug resistance in tumour cells.

Many studies have found that various members of the ABC superfamily play a role in mediating resistance to platinum compounds.^4^ The ABC transport system is a huge family, which includes seven subfamilies, namely ABCA, ABCB, ABCC, ABCD, ABCE, ABCF and ABCG.^5^, ^6^, ^7^ Some of them have been proven to be closely related to multidrug resistance, such as multidrug resistance proteins (ABCB1), multidrug resistance‐associated proteins (ABCC), and breast cancer resistance protein (ABCG2).^8^ The ABCC subfamily contains 11 subgroups encompassing ABCC1 to ABCC11. As a member of the ABCC subfamily, ABCC2 plays an important role in transporting endogenous and exogenous substances and impressing drug absorption, distribution and excretion.^9^ It has a protective effect on the body, and at the same time, the expression of ABCC2 is also closely related to the effect of chemotherapy on malignant tumours. ABCC2 has important clinical value in clinical chemotherapy and the evaluation of the effect of chemotherapy. For patients diagnosed with urothelial carcinoma of the urinary bladder, upon DDP treatment, DDP‐induced CCAAT/enhancer‐binding protein delta expression activated ABCB1 and ABCC2, and the high expression of ABCB1 and ABCC2 resulted in a decline in the effect of DDP.^10^ In ovarian cancer, upregulation of miR‐490‐3p enhanced the sensitivity of ovarian cancer cells to DDP by downregulating ABCC2 expression.^11^ In addition, upregulation of ABCC2 and ABCG2 transporters was observed in head and neck squamous cell carcinoma cell lines with cytoplasmically sequestered mutant p53, and these cell lines were frequently found to be more resistant to DDP.^12^, ^13^ ABCC2 inhibition of head and neck squamous cell carcinoma cells with MK571 markedly enhanced the sensitivity of HNSCC cells to DDP.^14^, ^15^, ^16^ Therefore, we found that the high expression of ABCC2 in various tumour cell lines is closely related to the resistance of these tumour cells to platinum drugs.

Our study compared normal A549 cells and DDP‐resistant cells (A549/DDP) using RNA sequencing. The results revealed that in A549/DDP cells, the expression levels of several ABCC family members were changed. ABCC2 and ABCC4 were upregulated, with ABCC2 exhibiting the most obvious change. After knocking down ABCC2 expression, the sensitivity of A549/DDP cells to DDP increased, and these effects were valid both in vivo and in vitro. Our study suggests that ABCC2 has potential as a therapeutic target for the treatment of patients with DDP‐resistant lung cancer.

## 2 MATERIALS AND METHODS

### 2.1 Cell culture

To establish the DDP‐resistant A549 cell subline, A549 cells were initially cultured in Dulbecco's modified Eagle's medium (DMEM) with an increasing dose of DDP for the first month (0.5, 1.0, 2.0 and 4.0 μM DDP increasing each week). In the following months, A549 cells were cultured in DMEM with DDP concentrations ranging from 4 to 10 μM. Ten μM DDP was then used to culture A549 cells for the following 2 months. After long‐term exposure, a subline resistant to DDP (referred to as A549/DDP) was successfully constructed. A549/DDP cells were maintained in DMEM supplemented with 1% penicillin/streptomycin and 10 μM DDP. The human non‐small cancer cell line A549 was purchased from the Chinese Academy of Sciences Cell Bank (Shanghai, China). A549 cells were cultured with 10% foetal bovine serum (FBS) and 1% penicillin/streptomycin.

### 2.2 Cell proliferation assay

Cell proliferation was assessed using the Cell Counting Kit‐8 (CCK‐8) assay. Specifically, A549 and A549/DDP cells (2000‐5000 cells/well) were seeded in 96‐well plates and cultured for 24, 48 and 72 hours. CCK‐8 solution (Beyotime, China) was added to the 96‐well plates and incubated for 1 hour. The absorbance (optical density, OD) at 450 nm was then determined to calculate cell proliferation viability.

### 2.3 Flow cytometric analysis of cell cycle distribution

A549 and A549/DDP cells (2 × 10^5^ cells/well) were seeded in 6‐well plates. For cell cycle distribution analysis, the cells were collected using centrifugation at 1500 rpm for 5 minutes, and the cell precipitate was washed with 70% alcohol and stored overnight in 70% alcohol. The next day, the cell precipitation was centrifuged at 1500 rpm for 5 minutes, and the supernatant was removed. Cells were stained using a Cell Cycle Staining Kit (Beyotime, China) for 30 minutes in the dark and then analysed using flow cytometry.

### 2.4 TUNEL apoptosis assay

A549 and A549/DDP cells were seeded in confocal small dishes. Cells were fixed with 4% paraformaldehyde for 30 minutes, incubated with 0.1% Triton‐X PBS for 2 minutes at 4°C, and then covered with 50 μL of TUNEL (Beyotime, China) assay solution (2 μL of TdT enzyme with 48 μL of fluorescence‐labelling solution) per dish. The nuclei of early apoptotic cells were labelled in red. Positive cells were detected using fluorescence microscopy to determine the number of apoptotic cells.

### 2.5 RNA isolation and quantitative real‐time polymerase chain reaction

Total RNA was extracted from the cells using TRIzol reagent (Invitrogen). cDNA was obtained using a reverse transcription kit (Takara). Quantitative real‐time polymerase chain reaction (qRT‐PCR) was performed using SYBR Premix Ex Taq (Takara, Japan). GAPDH was used as the standard control. All fold changes were calculated using the relative quantification method (2^−ΔΔCt^). The following primers were used: ABCC2: forward 5′‐CTCACTTCAGCGAGACCG‐3′, reverse 5′‐CCAGCCAGTTCAGGGTTT‐3′; ABCG2: forward 5′‐ACCATTGCATCTTGGCTGTC‐3′, reverse 5′‐CGATGCCCTGCTTTACCAAA‐3′; ABCA1: forward 5′‐GATGGCAATCATGGTCAATGG‐3′, reverse 5′‐AGCTGGTATTGTAGCATGTYCCG‐3′; GAPDH: forward 5′AGGTCGGTGTGAACGGATTC‐3′, reverse 5′‐TGTAGACCATGTAGTTGAGGTCA‐3′.

### 2.6 Western blot assay

A549 and A549/DDP cells were lysed on ice for 30 minutes in lysate buffer (RIPA; Beyotime, Shanghai, China) with 1% phenylmethylsulfonyl fluoride (1 mM). The lysates were centrifuged at 12000 rpm at 4°C for 15 minutes. The supernatant was collected, and protein concentrations were determined using a bicinchoninic acid assay (KeyGen Biotech, Jiangsu, China). The supernatant was mixed with 5 × SDS at a 4:1 ratio and incubated at 100°C for 10 minutes. The proteins (20 μg) were then separated on SDS‐polyacrylamide gels (12% separation gels) and transferred to polyvinylidene fluoride (PVDF) membranes (Millipore, Burlington, Massachusetts). The PVDF membranes were blocked with 5% skim milk (skim milk dissolved in TBST) for 2 hours at room temperature. The primary antibody was ABCC2 (ab205718, 1:1000) antibody purchased from Abcam. Caspase‐3 #9662, PARP #9532, Cleaved Caspase‐3 #9664, and β‐actin #4970 were purchased from Cell Signalling Technology (dilution 1:1000).

### 2.7 shRNA transfection and establishment of stable cell lines

sh‐negative control, sh1‐ABCC2 and sh2‐ABCC2 were purchased from GenePharma (Shanghai, China). Each shRNA was packaged in 293 T cells for 24 hours and collected according to the manufacturer's instructions. All transfections were conducted using Lipofectamine 3000 Transfection Reagent (Invitrogen, Thermo Fisher, Carlsbad, California). Forty‐eight hours after transfection, puromycin was added to select stable cell lines for 1 month.

### 2.8 Animal experiment

Male BALB/c nude mice (4 weeks old) were purchased from the Beijing Vital River Laboratory Animal Technology Co. Mice were subcutaneously implanted with 5 × 106 A549 and A549/DDP cells (pretreated with sh‐control or sh1‐ABCC2). After 7 days of subcutaneous implantation, the tumour volume was measured every 4 days until day 20 (formula: volume = width^2^ × length/2; units: mm^3^). When the maximum tumour volume reached 100 mm^3^, 16 mice were treated with DDP (6.0 mg/kg, every 3 days) or saline (50 μL, every 3 days). The mice were sacrificed on day 20 and the tumours were removed. All procedures involving experimental animals were performed under the guidelines of the Animal Ethics Committee of Zhejiang Provincial People's Hospital.

### 2.9 Immunohistochemistry

To detect ABCC2 expression, mouse subcutaneous tumours were fixed with paraffin. Tissues were dewaxed and hydrated using xylene and ethanol After that, the antigens were recovered. Antigen retrieval was performed at 95°C for 20 minutes. Endogenous peroxidase activity was blocked with 3% hydrogen peroxide (H_2_O_2_). After incubating the primary and secondary antibodies, haematoxylin was used for counterstaining. Finally, the samples were dehydrated and made transparent, and the slides were blocked using resin. The main antibody, Ki‐67 (1.5 μg/mL, ab238020), was purchased from Abcam. The number of Ki‐67 positive cells was counted using an Olympus inverted microscope and cells were scored in eight randomly chosen fields under a magnification of ×200 per sample.

### 2.10 Statistical analysis

Data from the three independent experiments are expressed as the mean ± SD. Student's *t* test or one‐way analysis of variance (ANOVA) was used to compare means. The relevant data were analysed using GraphPad 6.0. Differences between samples were considered significant at *P* < .05.

## 3 RESULTS

### 3.1 Upregulation of ABCC2 is related to lung cancer prognosis

**FIGURE 1:**
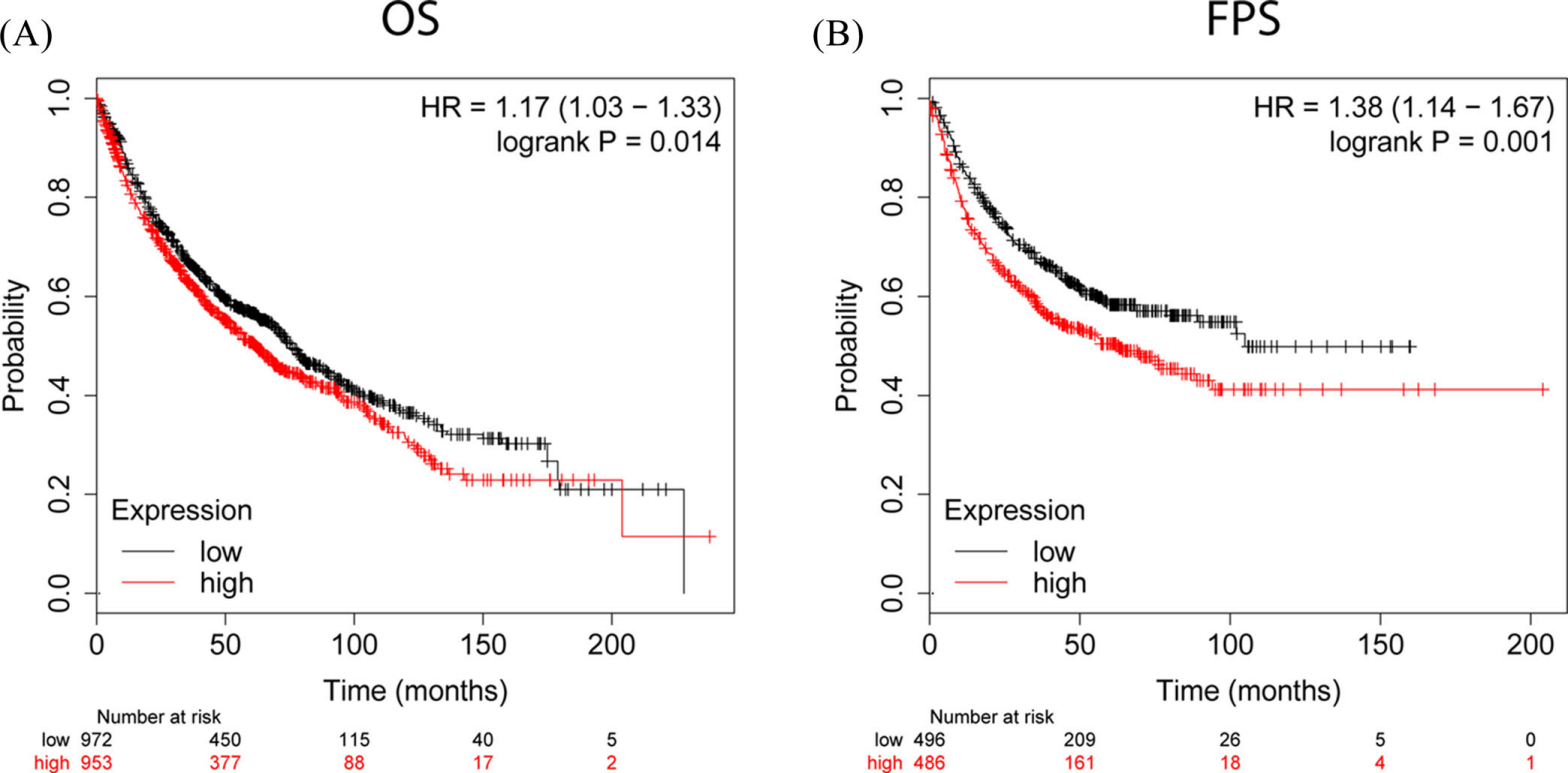
Upregulation of ABCC2 is related to lung cancer prognosis. Association between ABCC2 expression and OS, A, and PFS, B. OS, overall survival; PFS, progression‐free survival

We analysed the relationship between ABCC2 expression level and survival rate for patients with lung cancer using an online Kaplan‐Meier survival analysis tool (KMplot, http://kmplot.com/analysis/). The results showed that higher expression levels of ABCC2 were significantly associated with poor overall survival (OS) (*P* < .01) in lung cancer (1925 samples) (Figure 1A). Similarly, the increased expression of ABCC2 was significantly associated with progression‐free survival (PFS) (*P* < .001) in lung cancer (982 samples) (Figure 1B). These results suggest that higher levels of ABCC2 are associated with poor prognosis in lung cancer patients.

### 3.2 High expression of *ABCC2* gene increases resistance of A549/DDP cells to DDP

**FIGURE 2:**
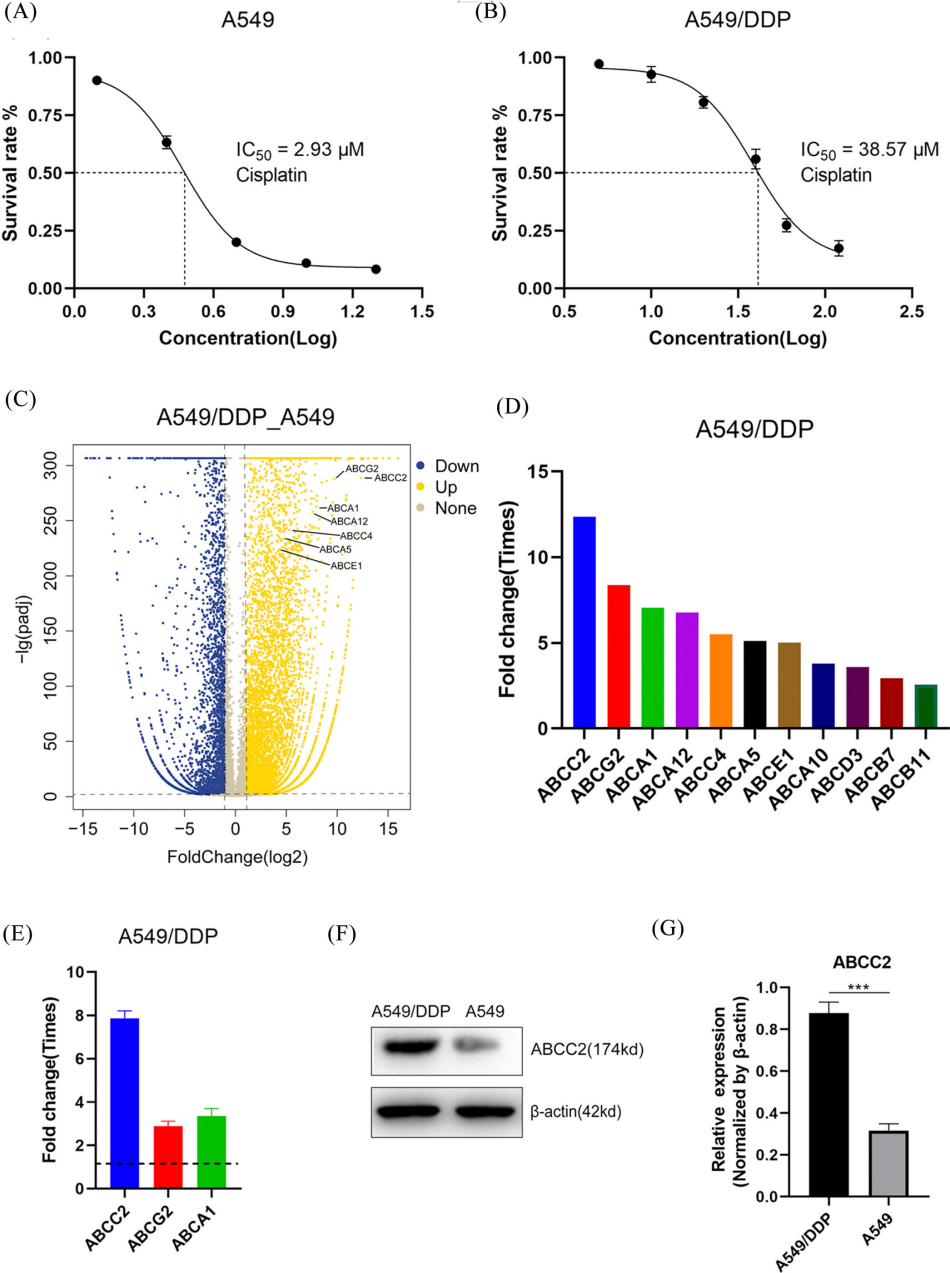
High expression of *ABCC2* gene increases resistance of A549/DDP cells to DDP. A,B, A549 and A549/DDP cells treated with DDP were measured using CCK8 after 48 hours. The IC_50_ in A549 cells and A549/DDP cells was 2.93 and 38.57 μM, respectively. The concentrations of DDP in A549 cells were 1.25, 2.5, 5, 10, 20 and 40 μM. The concentrations of DDP in A549/DDP were 5, 10, 20, 40, 60 and 120 μM. C,D, RNA sequencing showed that the expression of cell membrane transporter family related genes increased. Among them, expression of ABCC2 increased most significantly, reaching 12.351 times that of A549 cells. E, PCR results confirmed that a significant increase in ABCC2 expression was consistent with the RNA sequencing results. F, Western blot results showed that the expression of ABCC2 in DDP‐resistant cell lines also increased significantly at the protein level. G, Quantitative analysis of western blot in Fig. 2F. ***p < 0.001

A549 and A549/DDP cells were treated with different concentrations of DDP for 48 hours. The CCK‐8 results showed that the IC_50_ values of DDP in A549 and A549/DDP cells were 2.93 and 38.57 μM, respectively. The IC_50_ of A549/DDP cells was more than 10‐fold that of A549 cells. This demonstrated that we successfully constructed DDP‐resistant A549 cells (Figure 2A,B). The RNA sequencing results indicated that the expression level of the cell membrane transporter family increased in A549/DDP cells, with the greatest increases recorded in the expression of ABCC2 (12.531 times), ABCG2 (8.386 times), and ABCA1 (7.054 times) compared with A549 cells (Figure 2C,D). We then examined A549 and A549/DDP cells using PCR and found that the expression of ABCC2 (7.860 times) had increased significantly. Hence, the PCR results were consistent with the RNA sequencing data (Figure 2E). In addition, western blot analysis also revealed an increase in ABCC2 protein expression levels (Figure 2F,G). Therefore, in DDP‐resistant A549 cell lines, we found that ABCC2 exhibited higher expression in terms of both mRNA and protein levels as compared with that in normal A549 cells.

### 3.3 Knockdown of ABCC2 partially reversed the resistance of A549/DDP to DDP

**FIGURE 3:**
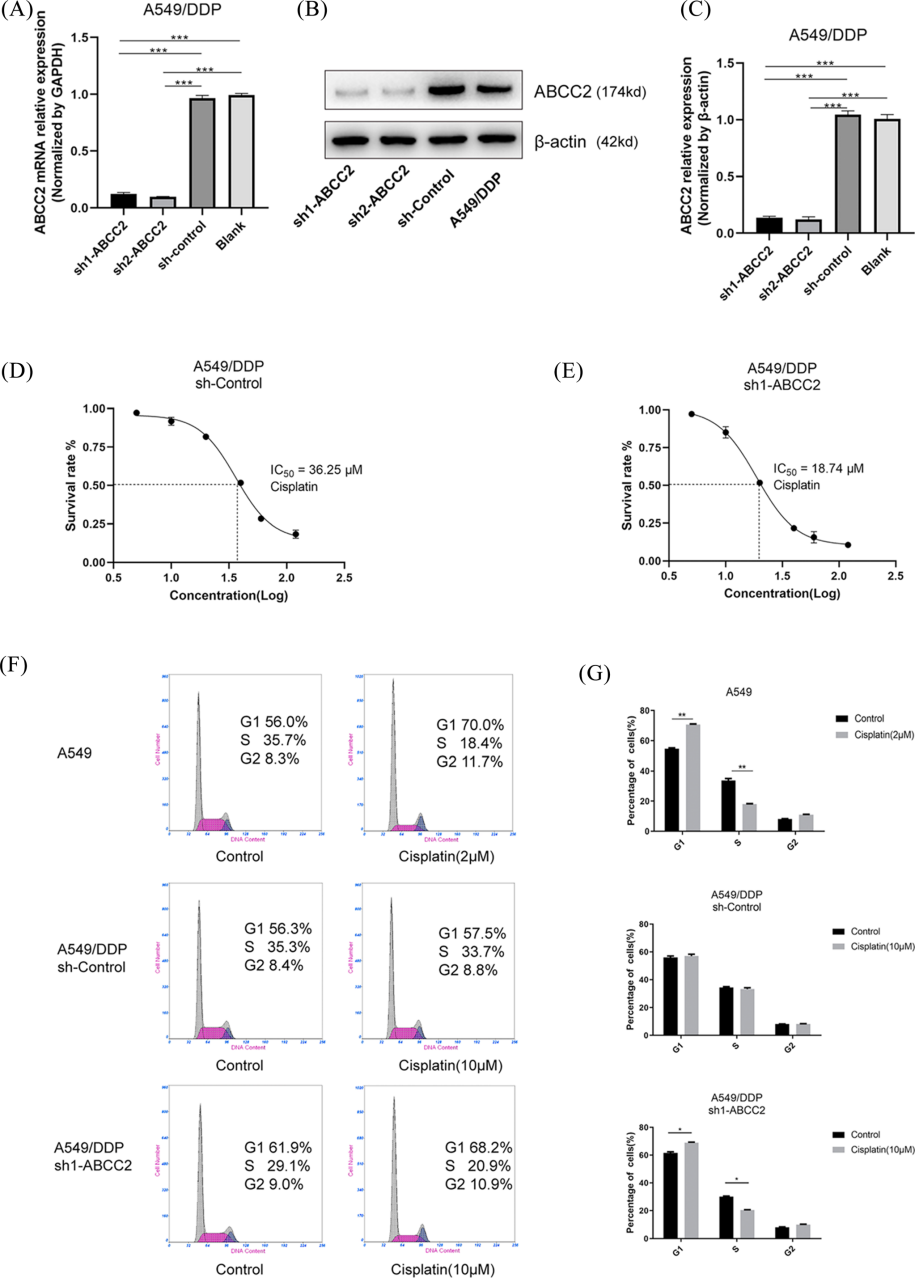
Knockdown of ABCC2 partially reversed the resistance of A549/DDP to DDP. A‐C, ABCC2 expression was analysed using qRT‐PCR and western blot assays after A549/DDP cells were transfected with sh1‐ABCC2, sh2‐ABCC2 and sh‐control. D,E, The IC_50_ in A549/DDP transfected with sh‐control or sh1‐ABCC2 were 36.25 and 18.74 μM, respectively. The concentrations of DDP in A549/DDP were 5, 10, 20, 40, 60, and 120 μM. F,G, A549 cells were treated with DDP (2 μM) for 48 hours. A549/DDP cells were treated with DDP (10 μM) for 48 hours. Cell cycle distribution assays were performed in A549 and A549/DDP cells using flow cytometry (n = 3, each group). *p < 0.05, **p < 0.01,***p < 0.001

Increased expression of ABCC2 in A549/DDP cells was revealed by PCR and western blot results. To investigate the effects of ABCC2 on DDP resistance, A549/DDP cells were transfected with sh‐control, sh1‐ABCC2 or sh2‐ABCC2. Transfection efficiency was determined using qRT‐PCR and western blotting. The results showed that ABCC2 expression significantly decreased after transfection in sh1‐ABCC2 and sh2‐ABCC2 groups (Figure 3A‐C). MTT experiments showed that the IC_50_ of DDP in the sh1‐ABCC2 group decreased to 18.74 μM compared with that in the sh‐Control group (36.25 μM). Therefore, the resistance to DDP in A549/DDP cells decreased with lower ABCC2 gene expression (Figure 3D,E). Flow cytometry analysis showed that after 48 hours of DDP treatment, the proportion of G1 phase cells increased, while the proportion of S phase cells decreased in the normal A549 group.

In the A549/DDP sh‐control group, DDP had no effect and the proportion of cells in each cell cycle was almost unchanged. On the other hand, in the A549/DDP sh1‐ABCC2 group, cell cycle changes were similar to those of A549 normal cells; the proportion of G1 phase cells increased and the proportion of S phase cells decreased (Figure 3F,G). The decreased number of cells in S phase indicated that most of the A549/DDP cells experienced cell cycle arrest in the G1 phase and could not enter the S phase, so they failed to complete DNA replication. In conclusion, ABCC2 knockdown reverses DDP resistance and promotes G1 phase arrest in A549/DDP cells.

### 3.4 Knockdown of ABCC2 promotes A549/DDP cell apoptosis by activating the caspase‐3 signalling pathway

**FIGURE 4:**
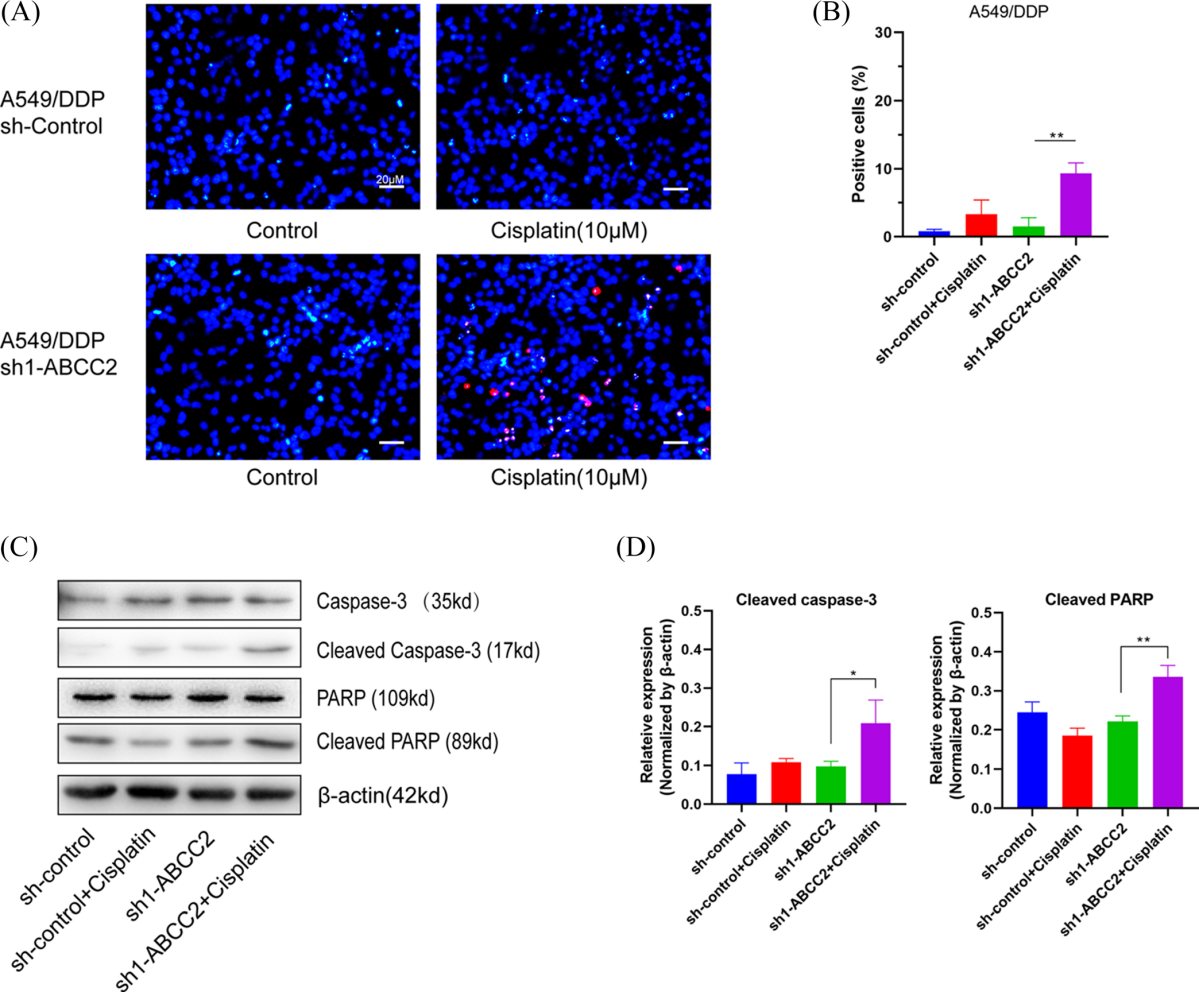
Knockdown of ABCC2 promotes A549/DDP cell apoptosis by activating the caspase‐3 signalling pathway. A,B, Apoptosis of A549/DDP cells was examined using TUNEL assay. Nuclei labelled with red fluorescence represent early apoptotic cells. The TUNEL results reveal that the apoptosis rate was increased in the sh1‐ABCC2 + DDP (10 μM) group (scale bar = 20 μm). C,D, A549/DDP sh‐control and sh1‐ABCC2 cells were treated with DDP (10 μM) for 48 hours. Western blot revealed that the expression of apoptosis‐related genes, cleaved caspase‐3 and cleaved PARP, was increased. *p < 0.05, **p < 0.01

TUNEL staining showed that the apoptosis rate of A549/DDP cells in the sh1‐ABCC2 group increased significantly (Figure 4A). The apoptosis rate in sh1‐ABCC2 cells was 9.5% ± 1.1%, while almost no apoptosis was observed in the other groups. PARP cleavage is considered an important indicator of apoptosis and an indicator of caspase‐3 activation. A protease became cleaved caspase‐3 when caspase‐3 was activated. Increased expression of cleaved PARP and cleaved caspase‐3 in the sh1‐ABCC2 + DDP group indicated that the caspase apoptosis signalling pathway was activated (Figure 4C,D).

### 3.5 ABCC2 inhibits tumour growth in vivo

**FIGURE 5:**
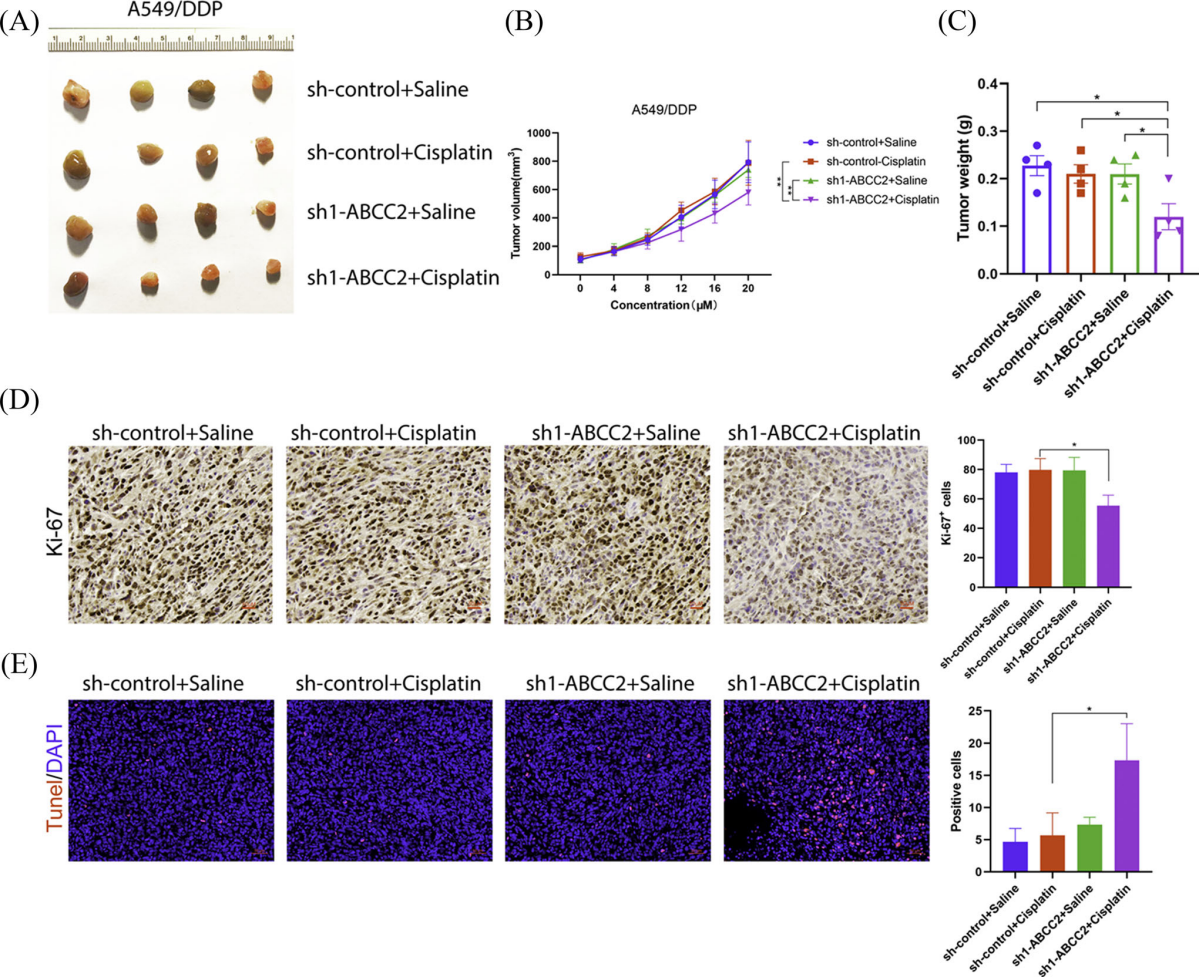
ABCC2 inhibits tumour growth in vivo. A, A549/DDP cell‐derived subcutaneous neoplasms on day 20 (four groups, n = 4). B, The volumes of the tumours were measured from day 0 to day 20 in each group (formula: volume = width^2^ × length/2; units: mm^3^). C, Tumour weights of A549/DDP xenografts were measured at day 20 after DDP treatment. D, The expression of Ki‐67 was detected using immunohistochemistry. scale bar = 50 μm. E, Apoptosis in subcutaneous tumours was detected using TUNEL staining. Scale bar = 50 μm. *p < 0.05

We have confirmed that DDP promotes apoptosis of A549/DDP cells transfected with sh1‐ABCC2 in vitro. Therefore, we injected A549/DDP cells into nude mice to establish a xenograft model. A549/DDP cells were transfected with sh‐control and sh1‐ABCC2. Nude mice were sacrificed on day 20, and the subcutaneous tumours were removed (Figure 5A). Tumour volumes were measured from day 0 to day 20 after DDP treatment. The tumour size of the sh1‐ABCC2 + DDP group was markedly smaller than that of the other groups (Figure 5B). Tumours were weighed after 20 days of tumour formation in nude mice. Similar to the tumour size results, the sh1‐ABCC2 + DDP group also had a lower tumour weight than the other groups (Figure 5C). In addition, the expression of Ki‐67 in the sh1‐ABCC2 + DDP group was significantly lower than that in the other groups according to immunohistochemical staining. TUNEL staining showed that apoptosis in subcutaneous tumours in the sh1‐ABCC2 + DDP group increased to a large extent (Figure 5D). The results of these in vivo experiments demonstrate that A549/DDP cells are more sensitive to DDP after knockdown of ABCC2.

## 4 DISCUSSIONS

Since DDP, the first platinum‐based drug, was launched in 1978, it has been widely used in the treatment of a variety of malignant solid tumours. These platinum‐based drugs enter the cells through the transmembrane and bind to DNA molecules, resulting in changes in DNA structure, and eventually DNA replication and transcription disorders, which cause cell apoptosis. Platinum drugs have long been used as the first choice of chemotherapy drugs for patients with lung cancer, ovarian cancer, and colon cancer after surgery, prolonging the life of patients. However, platinum drugs are subject to some restrictions. The limiting factors include primary or secondary tumour resistance, and serious adverse reactions, such as nephrotoxicity, gastrointestinal reaction, ototoxicity, neurotoxicity and renal toxicity, which are the most important dose‐limiting toxicities. In order to find safer and more effective chemotherapeutic drugs, in the past 30 years, two (carboplatin and oxaliplatin) were selected from 23 platinum antitumor drugs and gained approval in the international market. Several platinum‐based antitumor drugs (Nedaplatin, Loplatin and Heptinplatin) are marketed in a few countries. To date, there are four platinum antitumor drugs (Setplatin, Picoplatin, Lipoplatin and ProLindac) in different clinical studies. However, with continuous treatment with chemotherapeutic drugs, drug resistance in tumour cells is an unavoidable phenomenon.

Previous research has shown that ATP‐binding cassette (ABC) transporters play important roles in drug chemoresistance in many cancers.^17^ ABCC2, a member of the ABC family of transmembrane proteins, is also known as multidrug resistance‐associated protein 2 (MRP2) or the canalicular multiple organic anion transporter (cMOAT).^7^, ^18^ ABCC2 is mainly present in the outer membrane surrounding liver cells, and within small amounts in the kidneys, intestines and placenta.^19^, ^20^, ^21^, ^22^ After the long‐term use of platinum drugs in patients with ovarian cancer, lung cancer and colon cancer, the ABCC2 gene is often upregulated in some parts of these patients and upregulation of the ABCC2 gene usually causes patients to develop resistance to platinum drugs.^23^, ^24^ In addition, high ABCC2 expression is correlated with poor overall survival. ABCC2 has also been shown to increase DDP resistance and is controlled by let‐7c in NSCLC.^25^, ^26^, ^27^

In our study, we demonstrated that ABCC2 was upregulated in DDP‐resistant cells (A549/DDP). RNA sequencing results showed that the expression levels of various proteins related to cell membrane transporters were upregulated, including ABCC2 and ABCC4, and the expression of ABCC2 showed the most significant increase. Next, we performed PCR on A549/DDP and A549 cells, and changes in ABCC2 mRNA expression levels were consistent with the RNA sequencing data. The cell cycle results showed that normal A549 cells accumulated in the G1 phase after DDP treatment, which caused DNA replication disorders and eventually led cells to enter the apoptotic phase. However, this phenomenon was not observed in the A549/DDP group, in which DDP lost its original role in damaging the DNA of A549/DDP cells. When shRNA was used to knock down the expression of ABCC2, the effect of DDP on the cell cycle partially recovered and the cells in the DDP‐treated group stagnated in the G1 phase. Comparing normal A549 cells and drug‐resistant A549/DDP cells, the increase in the expression of the related membrane transporters blocked the transmembrane transport of cells, causing A549/DDP cells to acquire high resistance to DDP. Similarly, knockdown of ABCC2 in A549/DDP cells reversed cell resistance to DDP in vivo.

Collectively, these results indicate that the reduced expression of ABCC2 is likely to restore DDP sensitivity in DDP‐resistant NSCLC cancer cells. Our results highlight that targeting ABCC2 is considered as an approach to reverse DDP resistance in NSCLC patients.

## CONFLICT OF INTEREST

The authors declare no conflicts of interest.

## Data Availability

The datasets generated during the current study are available from the corresponding author on reasonable request.

## References

[1] Behera M , Pillai RN , Owonikoko TK , et al. Bevacizumab in combination with taxane versus non‐taxane containing regimens for advanced/metastatic nonsquamous non‐small‐cell lung cancer: a systematic review. J Thorac Oncol. 2015;10(8):1142‐1147. 26200267 10.1097/JTO.0000000000000572

[2] Paolicchi A , Sotiropuolou M , Perego P , et al. Gamma‐glutamyl transpeptidase catalyses the extracellular detoxification of cisplatin in a human cell line derived from the proximal convoluted tubule of the kidney. Eur J Cancer. 2003;39(7):996‐1003. 12706370 10.1016/s0959-8049(03)00067-4

[3] Heyza JR , Arora S , Zhang H , et al. Targeting the DNA repair endonuclease ERCC1‐XPF with green tea polyphenol epigallocatechin‐3‐gallate (EGCG) and its prodrug to enhance cisplatin efficacy in human cancer cells. Nutrients. 2018;10(11):1644. 10.3390/nu10111644 PMC6267282 30400270

[4] Baiceanu E , Crisan G , Loghin F , Falson P . Modulators of the human ABCC2: hope from natural sources? Future Med Chem. 2015;7(15):2041‐2063. 26496229 10.4155/fmc.15.131

[5] Ito K , Oleschuk CJ , Westlake C , Vasa MZ , Deeley RG , Cole SP . Mutation of Trp1254 in the multispecific organic anion transporter, multidrug resistance protein 2 (MRP2) (ABCC2), alters substrate specificity and results in loss of methotrexate transport activity. J Biol Chem. 2001;276(41):38108‐38114. 11500505 10.1074/jbc.M105160200

[6] Bruhn O , Cascorbi I . Polymorphisms of the drug transporters ABCB1, ABCG2, ABCC2 and ABCC3 and their impact on drug bioavailability and clinical relevance. Expert Opin Drug Metab Toxicol. 2014;10(10):1337‐1354. 25162314 10.1517/17425255.2014.952630

[7] Colombo S , Soranzo N , Rotger M , et al. Influence of ABCB1, ABCC1, ABCC2, and ABCG2 haplotypes on the cellular exposure of nelfinavir in vivo. Pharmacogenet Genomics. 2005;15(9):599‐608. 16041239 10.1097/01.fpc.0000172241.42546.d3

[8] Hoffmann U , Kroemer HK . The ABC transporters MDR1 and MRP2: multiple functions in disposition of xenobiotics and drug resistance. Drug Metab Rev. 2004;36(3–4):669‐701. 15554242 10.1081/dmr-200033473

[9] Pedersen JM , Khan EK , Bergstrom CAS , Palm J , Hoogstraate J , Artursson P . Substrate and method dependent inhibition of three ABC‐transporters (MDR1, BCRP, and MRP2). Eur J Pharm Sci. 2017;103:70‐76. 28263911 10.1016/j.ejps.2017.03.002

[10] Wang WJ , Li CF , Chu YY , et al. Inhibition of the EGFR/STAT3/CEBPD axis reverses cisplatin cross‐resistance with paclitaxel in the urothelial carcinoma of the urinary bladder. Clin Cancer Res. 2017;23(2):503‐513. 27435393 10.1158/1078-0432.CCR-15-1169

[11] Tian J , Xu YY , Li L , Hao Q . MiR‐490‐3p sensitizes ovarian cancer cells to cisplatin by directly targeting ABCC2. Am J Transl Res. 2017;9(3):1127‐1138. 28386339 PMC5376004

[12] Tonigold M , Rossmann A , Meinold M , et al. A cisplatin‐resistant head and neck cancer cell line with cytoplasmic p53(mut) exhibits ATP‐binding cassette transporter upregulation and high glutathione levels. J Cancer Res Clin Oncol. 2014;140(10):1689‐1704. 24913304 10.1007/s00432-014-1727-y PMC11823790

[13] Teft WA , Winquist E , Nichols AC , et al. Predictors of cisplatin‐induced ototoxicity and survival in chemoradiation treated head and neck cancer patients. Oral Oncol. 2019;89:72‐78. 30732962 10.1016/j.oraloncology.2018.12.010

[14] Guminski AD , Balleine RL , Chiew YE , et al. MRP2 (ABCC2) and cisplatin sensitivity in hepatocytes and human ovarian carcinoma. Gynecol Oncol. 2006;100(2):239‐246. 16213010 10.1016/j.ygyno.2005.08.046

[15] Grover S , Kukreti R . A systematic review and meta‐analysis of the role of ABCC2 variants on drug response in patients with epilepsy. Epilepsia. 2013;54(5):936‐945. 23506516 10.1111/epi.12132

[16] Comsa E , Nguyen KA , Loghin F , et al. Ovarian cancer cells cisplatin sensitization agents selected by mass cytometry target ABCC2 inhibition. Future Med Chem. 2018;10(11):1349‐1360. 29848100 10.4155/fmc-2017-0308

[17] Xue T , Lu ZN . Association between the polymorphisms in the ATP‐binding cassette genes ABCB1 and ABCC2 and the risk of drug‐resistant epilepsy in a Chinese Han population. Genet Mol Res. 2016;15(4):gmr15048752. 10.4238/gmr15048752 27886333

[18] Chew SC , Singh O , Chen X , et al. The effects of CYP3A4, CYP3A5, ABCB1, ABCC2, ABCG2 and SLCO1B3 single nucleotide polymorphisms on the pharmacokinetics and pharmacodynamics of docetaxel in nasopharyngeal carcinoma patients. Cancer Chemother Pharmacol. 2011;67(6):1471‐1478. 21468756 10.1007/s00280-011-1625-9

[19] Genvigir FDV , Nishikawa AM , Felipe CR , et al. Influence of ABCC2, CYP2C8, and CYP2J2 polymorphisms on tacrolimus and mycophenolate sodium‐based treatment in Brazilian kidney transplant recipients. Pharmacotherapy. 2017;37(5):535‐545. 28316087 10.1002/phar.1928

[20] Zucchi S , Corsi I , Luckenbach T , Bard SM , Regoli F , Focardi S . Identification of five partial ABC genes in the liver of the Antarctic fish *Trematomus bernacchii* and sensitivity of ABCB1 and ABCC2 to Cd exposure. Environ Pollut. 2010;158(8):2746‐2756. 20627496 10.1016/j.envpol.2010.04.012

[21] Milanova A , Pavlova I , Yordanova V , Danova S . Effect of doxycycline and lactobacillus probiotics on mRNA expression of ABCC2 in small intestines of chickens. Iran J Vet Res. 2016;17(4):265‐267. 28224011 PMC5309459

[22] Neumanova Z , Cerveny L , Ceckova M , Staud F . Interactions of tenofovir and tenofovir disoproxil fumarate with drug efflux transporters ABCB1, ABCG2, and ABCC2; role in transport across the placenta. Aids. 2014;28(1):9‐17. 24413260 10.1097/QAD.0000000000000112

[23] Lee SH , Kim HJ , Lee JS , Lee IS , Kang BY . Inhibition of topoisomerase I activity and efflux drug transporters' expression by xanthohumol from hops. Arch Pharm Res. 2007;30(11):1435‐1439. 18087812 10.1007/BF02977368

[24] Zhang W , Shannon WD , Duncan J , Scheffer GL , Scheper RJ , McLeod HL . Expression of drug pathway proteins is independent of tumour type. J Pathol. 2006;209(2):213‐219. 16508919 10.1002/path.1955

[25] Au A , Baba AA , Azlan H , Norsa'adah B , Ankathil R . Clinical impact of ABCC1 and ABCC2 genotypes and haplotypes in mediating imatinib resistance among chronic myeloid leukaemia patients. J Clin Pharm Ther. 2014;39(6):685‐690. 25060527 10.1111/jcpt.12197

[26] Balasubramaniyan N , Devereaux MW , Orlicky DJ , Sokol RJ , Suchy FJ . Up‐regulation of miR‐let7a‐5p leads to decreased expression of ABCC2 in obstructive cholestasis. Hepatol Commun. 2019;3(12):1674‐1686. 31832574 10.1002/hep4.1433 PMC6887930

[27] Zhan M , Qu Q , Wang G , Zhou H . Let‐7c sensitizes acquired cisplatin‐resistant A549 cells by targeting ABCC2 and Bcl‐XL. Pharmazie. 2013;68(12):955‐961. 24400442

